# Clinical, Histopathological, Imaging, and Treatment Perspectives of Inflammatory Granulomatous Mastitis: Review of the Literature

**DOI:** 10.5152/eurasianjmed.2022.22306

**Published:** 2022-12-01

**Authors:** Fatih Alper, Hasan Abbasguliyev, Sevilay Özmen, Ahmet Yalçin, Bahar Yılmaz Çankaya, Müfide Nuran Akçay

**Affiliations:** 1 Department of Radiology, Atatürk University Faculty of Medicine, Atatürk University Research Hospital, Erzurum, Turkey; 2 Department of Pathology, Atatürk University Faculty of Medicine, Atatürk University Research Hospital, Erzurum, Turkey; 3 Department of General Surgery, Atatürk University Faculty of Medicine, Atatürk University Research Hospital, Erzurum, Turkey

**Keywords:** Breast, ultrasonography, mastitis, granulomatous mastitis, steroids

## Abstract

Inflammatory granulomatous mastitis is a benign inflammatory disease of the breast mostly presenting in puerperal women. The disease is characterized by recurrent bouts of mastitis with clinical picture of hyperemia, breast mass, and swelling of the breast with or without purulent discharge depending on the severity of the underlying inflammatory process. Although no true prevalence and incidence have been reported in the literature, there are several reported studies setting forth a predilection in specific ethnic groups and/or geographical areas. Due to the intricate nature of the disease, quite often inflammatory granulomatous mastitis may be mistaken for malignant processes of the breast and even so, there are no pathognomonic imaging appearances to differentiate one from another. The histopathological analysis is a definite way of diagnosis. In this article, we review the imaging manifestations and clinical and histopathological findings along with current trends of available treatment options in the literature and briefly discussed our institutional perspective regarding grading of inflammatory granulomatous mastitis based on ultrasonographic appearances.

**Main Points**

- To review the clinical and demographic distribution of inflammatory granulomatous mastitis.
- To review current pearls and pitfalls on imaging appearances of inflammatory granulomatous mastitis.
- To report our institutional anecdotal experience on ultrasonographic clinical grading of inflammatory granulomatous mastitis.
- To review briefly the diagnostic workup of inflammatory granulomatous mastitis based on histopathological analysis.
- In a nutshell, to review treatment trends on therapeutic modalities of inflammatory granulomatous mastitis.

## Introduction

Idiopathic granulomatous mastitis (IGM) is a benign inflammatory disease of the breast affecting most frequently women of childbearing age with a history of breastfeeding. The term was first used by Kessler and Wolloch^1^ in 1972, and despite advancements in imaging and therapeutic management strategies of breast lesions, IGM still lacks a standardized workup for diagnosis. There are several entities causing granulomatous breast lesions that should be excluded before diagnosing definite IGM.^2^ Currently no definite data are available for the exact incidence and prevalence except for some reported case series emphasizing dependence on geographic distribution.

Idiopathic granulomatous mastitis is presented with a plethora of clinical findings including pain, inflammation, hyperemia, and mass, the latter being the most common of all. Generally, patients present with clinical manifestations and physical examination findings mimicking infectious mastitis or inflammatory carcinoma. Most of the time, a multidisciplinary approach is sought to narrow down differentials and the imaging has a paramount effect in this scenario both as a preliminary evaluation and performing image-guided biopsies for further characterization of the lesion.

In this article, we are presenting state-of-the-art management strategies for evaluation, imaging, and therapeutic approaches that predominate in the literature as well as our own approach in ultrasonographic imaging along with proposed clinical staging and therapeutic modalities that are being applied at our home institution, Ataturk University Research Hospital, Department of Radiology.

## Demographics

Granulomatous mastitis (GM) disproportionately affects a minority of women at childbearing age,^3^ which has drawbacks in terms of reflecting true demographic distribution. Even though the true prevalence of IGM is not well established, in a report by U.S. Center for Disease Control, the yearly prevalence of GM was estimated to be 2.4/100 000 women in Indianapolis, between 2006 and 2008.^4^ Baslaim et al's^5^ retrospective analysis reported IGM in 20 cases out of 1106 benign breast lesions (1.8%), of which Saudi ethnicity was dominating with 75%. Despite worldwide commonality, there is a predilection for the disease in some ethnic populations.^6-7^ In a systematic review by Martinez-Ramos et al.^8^ among included 70 articles, Turkey was a leading country having more publications in regard to IGM than others. An association between IGM and the Turkish population was depicted in a recent scoping analysis by Metanat et al.^9^ involving 7161 patients from 34 different countries in both criteria—countries where most publications originated (29.4%) and the number of patients within papers (39.1%). With regards to the distribution of ethnicity in these studies, it should be inferred that only few studies/countries reported such distribution, and Hispanics (65.9%) followed by Chinese (12%) and African-American (5.8%) were the pioneers among reported ones. However, the anecdotal experience of our institution shows that Turks followed by Caucasian predominate in ethnical distribution within Turkey.

In a few reported studies, a predilection of IGM formation in parous women has been established. And in most patients, IGM shows up 2 months through 20 years following either pregnancy or lactation.^10-12^ In a study by Altintoprak et al.^14^ among 26 IGM-detected patients despite the lack of prepregnancy complaints, only 15.3% had a disease on nonlactating breast; however, no statistically significant associations could be established.

In our department, patients who were treated for biopsy-proven IGM between 2017 and 2022, all women had a pregnancy history of at least 1-2 years prior to disease inception and almost invariably the non-lactating breasts were affected the most. Even though the concept of nonlactating breast involvement was identified as a common risk factor in lactational mastitis, ductal ectasia, and periductal mastitis, such correlation was not elucidated in IGM as a primer factor.^13^

## Clinical Manifestations

The most common presenting clinical signs and symptoms of IGM in affected women are unilateral firm and tender palpable mass(es) with erythematous skin changes and/or fistulous tracts accompanied by purulent discharge. A “peau d’orange” appearance may present on physical examination which most of the time alerts clinicians for a possible underlying malignancy. The size of the lesion is variable ranging from 1 to 10 cm with or without debilitating pain on the affected side.^14^ Axillary lymphadenopathy is detected every now and then, if present, more concerning regarding possible breast malignancy.

The nipple–areolar complex is seldom affected, and accompanying findings such as nipple discharge, retraction, ulceration, and may present. Another manifestation of IGM is a uni- or multifocal abscess formation during the course of the disease.^15-17^ In these cases, care should be taken not to hesitate for biopsy if the clinical index of suspicion is high enough as inflammatory breast carcinomas may also present with such clinical findings. The clinical presentation of IGM is not homogeneous and may present with a plethora of findings that further preclude straightforward diagnosis.

**Figure 1.**
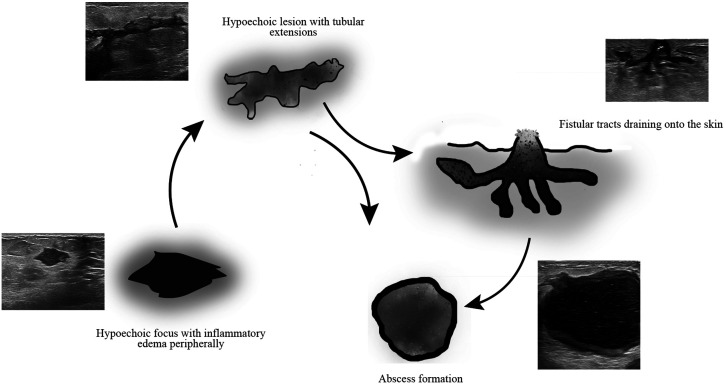
Ultrasonographic and illustrative appearances of biopsy-proven idiopathic granulomatous mastitis in a 32-year-old woman with complaints of right-side breast lump at presentation without past medical history of breast disease. In a clockwise starting from the left lower corner depicting the evolution of progression of the lesion within 2 months due to a delay in biopsy as well as treatment that has been denied by the patient at first show up. Core-needle biopsy was performed from the abscess following a short course of peroral antibiotic treatment.

The above withstanding, authors believe that there is a roadmap in the evolution of clinical manifestations of IGM along with respective ultrasonographic changes based on retrospective analysis (2017–2022) of biopsy-proven IGM patients at our home institution. The suggested clinical grading is as follows (Figure 1):

- On physical examination, indurated firm palpable mass with or without skin changes such as erythema and/or “peau d’orange” is being observed. On ultrasonography (USG), these changes are evidenced as non-uniform, sometimes circumscribed, heterogeneous hypoechoic mass(es) with diffuse hyperemia on Doppler examination.
- If goes untreated, lesion(s) progresses further involving large areas of skin changes. On ultrasonographic imaging either irregular hypoechoic masses with tubular extensions or less frequently, non-mass forming parenchymal distortion is being examined.
- Characterized by a purulent discharge which is depicted on imaging as sinus or fistular tracts. And at this stage, abscess formations are more pronounced. Even though nipple–areolar complex involvement indicates the severity of the disease, it should be noted that the disease may start from this region well.
- As a sequela of these changes, diffuse/local hypoechoic foci without vascular flow and alleviated subcutaneous hyperemia are characterized. This grade is the final stage observed following treatment.

## Pathophysiology

As the name implies, the idiopathic nature of the disease is attributable to the fact that granulomatous reactions are present in the absence of known stimuli. However, it is believed that some “environmental stimuli,” most often than not, initiate an inflammatory granulomatous reaction in genetically predisposed subjects. In our opinion, this hypothesis is plausible considering a high association of presentation of IGM in specific ethnic groups and/or geographic locations as well as predominating more in low socioeconomic populations.^18^

Although no precise causative factor was elucidated in the development of IGM, several triggers have been proposed, but neither of them was verified per se. Three main hypotheses have been predicated to explain IGM: autoimmune genesis, hormonal/lactational disorders, and infectious diseases.

The presence of instigating damage to intramammary ductal epithelium brings about a cascade of events starting with the migration of macrophages and lymphocytes to the inflammatory area ultimately transforming into granuloma tissue characterized by nodular aggregates.^14^ Several factors such as pregnancy, lactation, trauma, autoimmune disorders, diabetes, smoking, oral contraceptive usage, and α1-antitrypsin deficiency are considered culprit in disease commencement.^19^

### Imaging Spectrum

The acceptable approach for any palpable lump mass, as with IGM, for a patient younger than 40 years of age is USG and for patients above the age of 40 mammography is indicated.^20^ Additionally, new advanced techniques, elastography and tomosynthesis, have been incorporated into routine clinical practice. Magnetic resonance imaging (MRI) is of great value in cases when both USG and mammography are inconclusive or where remnant active tissue evaluation may be hindered by edematous tissue or in assessing the extent of disease involvement.

### Ultrasonographic Appearance

Ultrasonography is almost always performed as a primary mode of imaging modality in all patients, irrespective of age, presenting with an indication of mastitis. Also, USG is indicated for follow-up, imaging-guided biopsies, as well as image-guided intralesional therapies.

**Figure 2. A-E.**
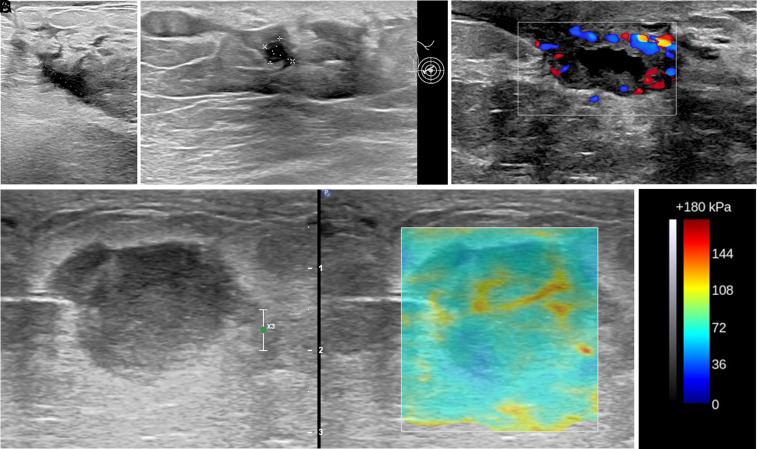
Targeted ultrasound images from (A) through (D) depicting different grades of idiopathic granulomatous mastitis (IGM) on different patients. (A) A fistula tract shows the drainage of purulent content on the background B-mode image and color Doppler map displays ongoing hyperemia within the wall of the tract. This finding corresponds to Grade II. (B) Hypoechoic solid-like mass measuring about 3.4 cm on long axis with diffuse increment on echogenicity of surrounding tissue obtained at gray-scale ultrasound at 9 o’clock position of the right breast in a 28-year-old postpartum woman presenting with denial of breastfeeding by her baby on the affected breast. Following 3 weeks of conservative treatment due to a lack of symptom resolution with progression in the size of the lesion, a biopsy was performed, and the result was IGM. This lesion demonstrates the earliest course of disease initiation and corresponds to grade I. (C) In another patient with a history of periductal mastitis on the contralateral breast prior 2.5 years was referred to the tertiary university hospital for further work-up of the lesion on the right side. The abscess formation (measurement markers) is clearly depicted within the extremely IGM-involved periareolar breast side. These findings correspond to grade III. (D) Elastosonographic image of the biopsy-proven IGM lesion. Color box (E) indicates the stiffness of tissue based on elasticity properties of the region of interest with red depicting the hardest and blue the softest.

Even though the USG appearance of IGM varies greatly, the most frequently reported finding is irregularly shaped hypoechoic mass(es) with or without tubular extensions.^11,16,21-24^ The tubular extension of the lesion is a descriptive term for the interconnected meshwork-like appearance of disease insinuating around lobules. From our own experience, this appearance along with ancillary findings including focal or diffuse skin thickening, subcutaneous or perilesional edema, increased echogenicity in subcutaneous fat (secondary to inflammation), and axillary reactive lymph node changes represent the early course of the disease, particularly grade II clinical staging (Figure 2A).

Our hypothesis is that an inciting lesion first starts as a non-uniform, sometimes circumscribed, heterogeneous hypoechoic mass, that is, grade I clinical staging, which evolves later into these hypoechoic tubular extensions^21,22^ (Figure 2B). We surmise that the high likelihood of missing grade I findings of IGM may be attributable to the late admission to tertiary hospital settings for a thorough workup in diagnosis. That is because, due to conflicting nature of IGM being mistaken for general mastitis, patients undergo an empirical antibiotic therapy which alleviates symptoms for a short period of time eventually leading to lesion burnout.

The grade III clinical staging (Figure 2C) is particularly seen in patients not undergoing any treatment, and this stage is characterized by extensive fistular tracts and purulent discharge with accompanying uni- or multilocal hyperemic thick-walled fluid collections, that is, abscess(es).^22-24^ Even though most abscesses in the body are caused by infectious pathogens, mostly bacteria, the possible underlying mechanism of such collections in IGM may partly be explained by the body’s defensive reaction to immure the spread of inflammatory reactions. However, in patients undergoing treatment, abscess formations may be omitted and only fistular tracts could predominate this stage, which in part alleviates the symptoms of patients. It is worth mentioning that there is no clear-cut distinctive boundary between grades, and clinical manifestations of each grade may be presented in the same patient at the same time. And considering the paucity of clinical trials in the literature for better delineation of such findings as a distinct stage, while attaining a definite clinical grade, all available data including multimodality imaging parameters should be considered.

The latest clinical grade IV of IGM denotes the final stage of disease featuring a sequela of findings, such as heterogeneous hypoechogenic foci without any vascular flow evidenced either on power or color Doppler examinations. Often time than not, sinus or fistular tract remnants may persist quite some time after total remission and should not be mistaken as an ongoing inflammatory process. The main clue for reassurance is to check ancillary findings along with the vascular flow in this region. In cases of uncertainty, additional imaging modalities should be exploited.

### Elastosonographic Appearance

It is salutary to incorporate elastography examination in the workup of IGM imaging. There are several literature reports on the efficacy of elastography in discriminating IGM and breast malignancy.^25-27^

A newly developed ultrasonographic elastography technique named shear wave elastography (SWE) is based on generating shear waves via utilizing acoustic radiation force which induces deformity within tissues. And depending on the degree of the stiffness, color maps (electrograms) are generated depicting the interrelation between shear-wave velocity as well as shear modulus^28-30^ (Figure 2D). This method allows a more objective and quantitative evaluation of breast lesion, specifically differentiating benign from malignant masses. A recent study by Toprak et al.^31^ investigating the qualitative and quantitative role of SWE in differentiating IGM from invasive ductal carcinoma (IDC), found sensitivity and specificity of 89% and 84%, respectively.^31^

In the study by Yağcı et al.^32^ strain elastography method was used for assessment of its contribution in differentiating IGM from malignant lesions via analyzing receiver operating characteristic curves. The diagnostic performance of strain elastography for IGM was 96% in specificity and 87% in sensitivity with strain ratio values in IGM (1.5 ± 0.8) lower than malignant lesions (5.3 ± 5.2).

Aslan et al^33^ took the topic a little further and investigated the correlation between SWE findings with pre-treatment IGM severity, and no correlation was found between them. In our opinion, to show the value of SWE in the prediction of clinical responsiveness of IGM treatment, more research should be conducted.

### Mammographic Appearance

There is great variability in mammographic findings and no pathognomonic feature has been described for IGM; however, focal asymmetry is most encountered. In the literature, many other appearances, including irregularly shaped or obscured mass, have been described.^11,20,22^ Also, as with other lesions of the breast, the conspicuity of lesion detection decreased as the density of the breast increased.

Other described mammographic findings are axillary lymphadenopathy, focal or diffuse skin thickening secondary to inflammatory edematous changes.^11,20^ Of note, in contrast to inflammatory breast cancer, extensive skin involvement is seldom affected in IGM. However, it has been stated that ultrasound is superior to mammography in the detection of clinically undetected axillary adenopathy and skin thickening.^22^

### Magnetic Resonance Imaging Appearance

Magnetic resonance imaging is commonly reserved for specific scenarios where USG and mammography are unsatisfactory for decision-making or in cases for identifying lesion extension, aiding biopsy, and evaluating ancillary extramammary findings. Additionally, MRI is useful in the evaluation of suspicious residual inflammatory areas. Although MRI is used less frequently in comparison to USG and mammography, the sensitivity of this modality is comparable with high positive predictive value.^3,11,22,34-36^

**Figure 3. A-D.**
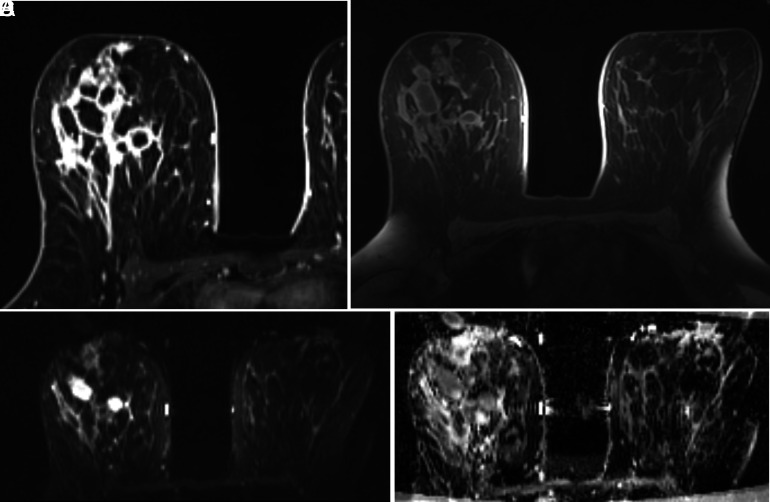
Idiopathic granulomatous mastitis (IGM) in a 24-year-old patient with a persistent history of IGM on the right breast for the past 3.2 years. A thorough evaluation was not yielding for any etiologic agent. The lesions on magnetic resonance imaging (MRI) show extensive segmental involvement with diffuse contrast enhancement on (A) flow dynamic and (B) post-contrast subtraction images. The mass shows restricted diffusion on (C), axially acquired image from diffusion-weighted MRI (*b *= 800 s/mm^2^) and a low apparent diffusion coefficient on (D). The patient underwent 5 courses of intralesional steroid injection therapy and no relapse was noted on 3 years of follow-up.

On MRI, either rim-enhancing lesion or, occasionally, heterogeneous non-segmental enhancing mass, or regional non-mass enhancement may be seen. Most frequently small rim-enhancing lesions demonstrate confluency on fluid-sensitive sequences, presumably indicating multifocal microabscesses (Figure 3). In general, skin changes may not be as conspicuous as we see on physical examination, but still focal or diffuse thickening with or without fistula tracts may be evidenced. In our experience, scrutinizing for solid components in cavitating lesions may be of great value as the size of these mural nodules could be used as a proxy for predicting treatment response.

## Histopathological Correlate

**Figure 4. A-C.**
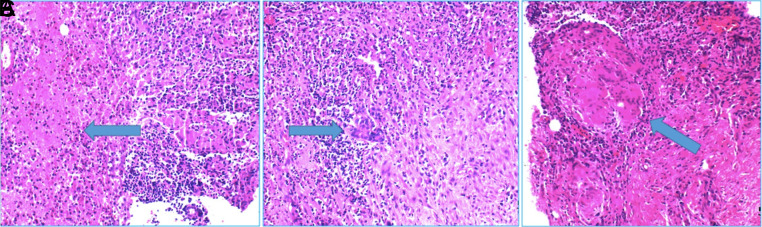
Images from A to C (blue arrows, H&E ×200 magnification) depict granuloma tissues on core-biopsy specimens.

On histopathologic examination, in the presence of granulomatous inflammation, the main objective should be to look for whether there is caseating necrosis or not, as the latter is a distinctive feature of IGM. The inflammatory changes are confined to ductal lobular units without affecting much major ducts or fat tissues.^37^ In the setting of chronic inflammation, aggregates of immune cells—multinucleated giant cells, plasma cells, and eosinophils—in and around lobules create granulomas. In the case of neutrophil-predominant granulomas, where microabscesses may form, cystic neutrophilic GM should be differentiated (Figure 4).^38^

**Figure 5. A-C.**
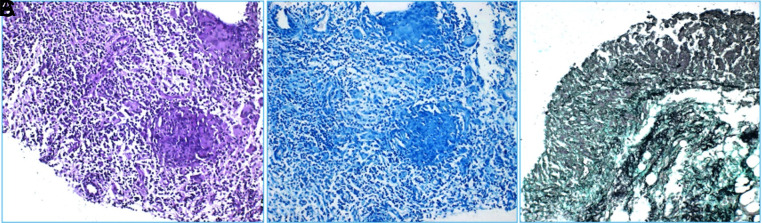
Non-necrotizing granulomas were negative for Periodic acid–Schiff (PAS) (A and B), Ziehl-Neelsen (EZN), and Grocott (C).

**Figure 6. A,B.**
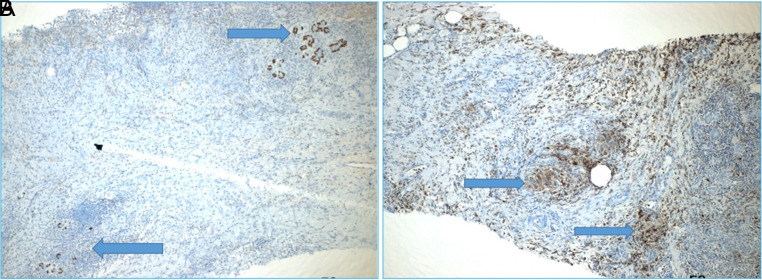
Immunohistochemically, P63 positive normal breast ducts (A—blue arrows) and granuloma structures formed by CD-68 positive epithelioid histiocytes (B—blue arrows).

A thorough analysis should be conducted on core-needle biopsy specimens to exclude any infectious causes or malignancy and diligently differentiate other causes of GM. Special microbial stains are utilized whenever granulomas are present. These are Ziehl-Neelsen (EZN) stain for acid-fast bacilli, periodic acid-Schiff for fungi^39^ (Figure 5). The usual reactivity of p63 staining immunohistochemically helps in ruling out malignancy. A positivity on CD68 stain is a characteristic of epithelioid histiocytes forming granulomas (Figure 6).^40^

## Treatment Strategies

Currently, the most accepted mode of treatment for debilitating IGM is surgical excision; however, even with surgery there is a chance of recurrence and for this very reason it is reserved as the last option. A growing number of publications have demonstrated that oral or local (or combined) steroid treatment may be of great efficacy either as an alleviative method prior to surgery or as a definite treatment option with low recurrence rates.^41-46^ Despite some unwanted side effects afflicted steroid use, currently no solid data are available regarding the time of termination of treatment and best-approached method is the complete clinical response.

In our institution, in between 2017 and 2019, loco-regional intravenous steroid injections at a dosage of 40 mg diluted in 0.9% NaCl solution were injected as an intralesional mode, and in patients with progressive course, combined therapy (oral + i.v.‚ ± topical steroid) was initiated. Since 2019 up until now, all patients with IGM admitted for i.v. steroid therapy, depending on the severity status of the disease—based on clinical and imaging parameters—starting dosage of 80 mg with intra- and perilesional administration has been preferred. Also, in patients presenting with abscesses, that is grade III clinical staging, aspiration of abscess preceding steroid injection is a preferable method. The main advantage of this method is that the systemic effects of steroids are being bypassed, multiple sessions may be applied, and the disease course is being visualized all along.

The above withstanding, in recent years a new trend of follow-up with conservative management, explicitly in mild cases, is encouraged. Still, there are studies reporting a high rate of recurrence with this approach.^47^

## Conclusion

Idiopathic granulomatous mastitis is a benign inflammatory disease of the breast affecting most frequently women of childbearing age with a history of breastfeeding.

Even though the true prevalence of IGM is not well established, the inclination to affect more Mediterranean, Iranian, and Asian women has been reported in the literature.

As the name implies, there is no definite causative factor, but several triggers, including autoimmunity, hormonal imbalance, and infectious agents, are implicated in pathophysiology.

Imaging modalities, particularly USG and mammography, play a vital role in preliminary analysis. The most common imaging finding is focal asymmetry and hypoechoic tubular extensions on mammography and USG, respectively. Advanced imaging modalities, including elastography and MRI, may occasionally increase diagnostic accuracy; however, none of these findings are pathognomonic and biopsy is indicated for a definite diagnosis.

The definite treatment of IGM remains controversial, but without medication or surgical treatment, the rate of persistence and natural course of recurrence eventually bring about lesion burnout down the road.
